# Optimizing Rooster Semen Preservation: Effect of Oxygen Exposure, Sample Rotation, and HEPES Buffer Supplementation

**DOI:** 10.3390/ani15162391

**Published:** 2025-08-14

**Authors:** Khomsan Buathalad, Thirawat Koedkanmark, Wuttigrai Boonkum, Vibuntita Chankitisakul

**Affiliations:** 1 Department of Animal Science, Faculty of Agriculture, Khon Kaen University, Khon Kaen 40002, Thailand; komsan.bua@kkumail.com (K.B.); thirawat.koe@kkumail.com (T.K.); wuttbo@kku.ac.th (W.B.); 2 Network Center for Animal Breeding and Omics Research, Khon Kaen University, Khon Kaen 40002, Thailand

**Keywords:** HEPES buffer, mechanical rotation, oxygen exposure, semen extender

## Abstract

This study aimed to investigate physical and biochemical strategies to optimize the preservation and fertilizing capacity of rooster semen during chilled storage and after artificial insemination (AI), respectively. Two semen extenders—0.9% sodium chloride (NaCl) and IGGKPh—were evaluated through two factorial experiments. In Experiment 1, a 2 × 2 factorial design was used to examine the effects of oxygen exposure (aerobic vs. reduced-oxygen conditions) and tube rotation (rotated vs. non-rotated) on semen quality during 24 h of storage at 5 °C. Sperm quality was evaluated based on progressive motility, viability, pH, and malondialdehyde (MDA) concentration. IGGKPh was significantly more effective than NaCl in preserving sperm function, maintaining motility above 70% and viability near 90%. Aerobic conditions and tube rotation improved motility but also increased MDA levels, indicating a tradeoff between enhanced metabolic activity and oxidative stress. Semen stored in NaCl lost its fertilizing capacity after 22 h, whereas IGGKPh under aerobic and rotated conditions resulted in significantly higher fertility rates (91.77%) compared with non-rotated samples. In Experiment 2, the effects of HEPES buffer supplementation (present vs. absent) and handling temperature (5 °C vs. 25 °C) were evaluated under simulated AI conditions. Semen extended in IGGKPh was stored at 5 °C for 22 h prior to handling, while NaCl samples were used immediately after dilution. Sperm quality was assessed at 0, 30, and 60 min of exposure. HEPES significantly reduced MDA levels and improved motility and viability in both extenders. Fertility rates were highest in the HEPES-supplemented groups, especially under chilled handling. In conclusion, optimal preservation of rooster semen requires a combination of metabolic support, adequate oxygen availability, gentle mixing, and pH stabilization. While IGGKPh was effective for storage up to 24 h, its performance was further enhanced by HEPES buffer. These findings offer practical recommendations for AI programs in poultry, particularly under field conditions where temperature fluctuations and delayed insemination are common.

## Simple Summary

Improving rooster semen preservation is essential for successful artificial insemination (AI), particularly in smallholder poultry production systems. This study explored ways to improve the short-term storage (up to 24 h) of Thai native rooster semen by examining how extender type, environmental conditions (oxygen exposure and mixing), and pH buffering affect sperm quality and fertility. Results showed that a simple saline solution (0.9% NaCl) was not suitable for storage beyond 12 h but could be used for very short periods if HEPES buffer was added and the temperature was kept at 5 °C. In contrast, the IGGKPh extender maintained sperm motility and viability for up to 24 h, especially when stored with oxygen and gentle rotation. The best fertility rate was observed in the IGGKPh group stored under those optimal conditions. These findings suggest that practical, low-cost strategies can improve AI outcomes in poultry, even in areas without access to advanced cooling or antioxidant systems.

## 1. Introduction

Artificial insemination (AI) is a key biotechnological tool that significantly enhances reproductive efficiency and accelerates genetic improvement in poultry production systems, particularly in economically important species such as turkeys and indigenous chicken breeds [1]. Although fresh semen can be used for AI, chilled semen is often preferred due to its advantages, including logistical flexibility and the facilitation of synchronized, batch inseminations. The effective use of chilled semen, however, depends on optimized storage protocols that preserve sperm viability and fertilizing potential. Rooster semen is typically stored at 2–5 °C in specialized extenders designed to maintain functional integrity for up to 24 h [2]. These extenders stabilize pH and osmolarity, supply essential metabolic substrates to support sperm function during cold storage. Commonly used extenders include IGGKPh [3], EK [4], BPSE [5], and NCAB [6].

To date, research has predominantly targeted biochemical modifications of extenders, such as antioxidant supplementation including plant extracts [7,8], and amino acids [6,9,10], as well as introduction of membrane stabilizers, to mitigate oxidative stress during storage [11]. In contrast, relatively little attention has been directed toward optimizing the physical and environmental conditions of semen storage, despite their potential to impact sperm physiology significantly. One critical yet underexplored factor is oxygen exposure within storage tubes. Studies in mammals, including stallions [12], boars [13], and rams [14], have shown that reducing the headspace air above semen samples preserves sperm viability by limiting the production of oxygen-driven reactive oxygen species (ROS). Excessive ROS promotes lipid peroxidation, DNA fragmentation, and protein oxidation, ultimately impairing sperm function and fertilization potential [15,16]. Avian sperm are particularly vulnerable to oxidative damage; however, the role of oxygen exposure during rooster semen storage remains unclear.

Another physical factor with possible relevance is the mechanical rotation of semen samples during storage. In boar semen, gentle rotation can prevent sperm sedimentation and promote sample homogeneity, contributing to improved motility and fertility outcomes [17]. This dynamic environment may also facilitate uniform nutrient distribution and stabilize pH. However, findings in mammals have been inconsistent, with some studies reporting no beneficial effects [18,19]. In addition, its application in avian semen storage has not yet been assessed.

Brief warming of semen during artificial insemination procedures, especially under tropical field conditions, also poses a practical challenge. Exposure to ambient temperatures can accelerate metabolic activity, increase oxidative byproducts, and disrupt extender buffering. HEPES buffer, a zwitterionic buffering agent known for its strong pH stability across temperature gradients, has demonstrated efficacy in mammalian extenders [20,21,22], but its effectiveness in poultry remains poorly documented.

While oxygen exposure, sample rotation, and HEPES buffering have been studied individually in other species, their combined or interactive effects on rooster sperm quality during chilled storage have not been elucidated. To address this gap, we designed a study using two extenders: commercial IGGKPh and 0.9% sodium chloride (NaCl). The latter is commonly used by Thai poultry farmers despite its limited buffering and nutritional properties, primarily due to its low cost, accessibility, and ease of preparation in field conditions [6]. This approach allowed us to evaluate both laboratory-grade and field-relevant solutions for semen preservation. Moreover, to validate these protocols under practical conditions, fertility outcomes were evaluated through artificial insemination trials in native chickens. This field-level assessment aligns with recent efforts to bridge laboratory findings with on-farm application in tropical poultry systems [6].

Accordingly, the present study aimed to evaluate the combined and individual effects of oxygen availability, sample rotation, and HEPES buffer supplementation on the quality and fertilizing capacity of chilled rooster semen. We hypothesized that these interventions would synergistically enhance sperm motility, viability, and fertility, particularly under field-relevant conditions.

## 2. Materials and Methods

### 2.1. Chemicals

All chemicals and reagents were obtained from Sigma-Aldrich Chemical Co. (St. Louis, MO, USA), unless otherwise specified. HEPES (4-(2-hydroxyethyl)-1-piperazineethanesulfonic acid; CAS No. 7365-45-9) was specifically used as a buffering agent in the semen extender formulations.

### 2.2. Animals and Management

This study used 40 Thai native Pradu Hang Dum roosters (32–40 weeks of age). The birds were housed individually in open-sided cages (48 × 45 × 45 cm) under natural photoperiod and temperature. Each rooster received a commercial diet (Balance 924, Betagro Co., Ltd., Bangkok, Thailand) at 130 g/bird/day, formulated to contain 17% crude protein, 3% crude fat, 6% crude fiber, and 13% moisture. Water was provided ad libitum.

For the fertility evaluation, 60 ISA Brown laying hens (32–40 weeks old) with laying performance above 80% were housed under similar conditions and fed the same diet at 120 g/hen/day. The same group of hens was used for repeated inseminations throughout the experimental period to reduce biological variation and minimize unnecessary animal use. Artificial insemination procedures were conducted once weekly throughout the experimental period.

All experimental procedures were approved by the Institutional Animal Care and Use Committee of Khon Kaen University (IACUC-KKU-7/68; Ref. No. 660201.2.31/55(6)) and adhered to the ethical guidelines for the care and use of animals as outlined by the National Research Council of Thailand.

### 2.3. Semen Sample Collection and Extender Preparation

Semen was collected twice weekly using the dorso-abdominal massage technique. Ejaculates were immediately deposited into 1.5 mL sterile microcentrifuge tubes pre-filled with 100 µL of either a 0.9% sodium chloride (NaCl) solution (KLEAN&KARE SALINE, A.N.B. Laboratories Co., Ltd., Bangkok, Thailand) or IGGKPh extender.

The IGGKPh extender was prepared according to the formulation of [3], containing 0.14 g potassium citrate monohydrate, 1.40 g sodium glutamate, 0.21 g sodium dihydrogen phosphate, 0.98 g disodium hydrogen phosphate, 0.90 g glucose, and 0.90 g inositol per 100 mL of deionized water. The final solution had a pH of 6.95 and an osmotic pressure of approximately 380 mOsm/kg.

Following collection, semen was gently mixed with the assigned extender, maintained at ambient temperature (22–25 °C), and transported to the laboratory within 20–30 min for initial evaluation of semen quality parameters.

### 2.4. Semen Evaluation and Dilution

Fresh semen samples were initially evaluated for mass movement using a compound light microscope (10× magnification; Olympus, Tokyo, Japan). A 3–5 µL aliquot was placed directly onto a clean glass slide without a coverslip. Mass movement was scored subjectively using a 0 to 5 scale, where 0 indicated no movement and 5 represented vigorous, wave-like motion with >90% forward progressive movement [8]. Only ejaculates with a volume exceeding 0.3 mL [23] and a motility score above 3.5 were pooled and assigned to the respective extender treatments.

Following pooling, the semen samples were evaluated for motility, viability, sperm concentrations, and pH, as follows:

- Sperm motility was assessed using phase-contrast microscopy at 40× magnification on a pre-warmed slide (37 °C). A 5–10 µL semen aliquot was mounted under a coverslip, and progressive motility (PMOT) was recorded. Mean PMOT values were 89.87 ± 1.91% for 0.9% NaCl and 93.14 ± 1.44% for IGGKPh;
- Sperm viability was determined using the eosin–nigrosine staining technique. A 5 µL semen aliquot was mixed with 20 µL of a pre-warmed staining solution (0.6% eosin and 5% nigrosine in distilled water), incubated for 1–3 min, smeared onto a glass slide, air-dried, and examined under oil immersion at 100× magnification. Viable sperm appeared unstained, while non-viable cells exhibited pink-stained heads, as determined by counting a minimum of 300 sperm cells per sample. Mean viability was 92.46 ± 0.45% for 0.9% NaCl and 95.20 ± 0.65% for IGGKPh;
- Sperm concentration was quantified using a hemocytometer. Semen was diluted 1:1000 in a 4% NaCl solution containing eosin to enhance cell visibility. A 10 µL aliquot of the diluted sample was loaded onto the hemocytometer and analyzed at 40× magnification. Mean sperm concentrations were 5.36 ± 0.51 × 10^9^ sperm/mL for 0.9% NaCl and 5.88 ± 0.95 × 10^9^ sperm/mL for IGGKPh;
- The pH of the semen was measured using a portable digital pH meter (HI98100 Checker Plus; Hanna Instruments, Woonsocket, RI, USA). Mean pH values were 7.05 ± 0.080 for 0.9% NaCl and 6.87 ± 0.007 for IGGKPh.

After quality assessment, pooled semen was diluted with each extender at a 1:3 (v/v) ratio according to experimental treatment assignments. The final concentration was standardized to approximately 100–150 × 10^6^ sperm per insemination dose. Diluted semen samples were cooled gradually from 25 °C to 5 °C over 1 h and subsequently stored at 5 °C in a laboratory refrigerator (Model GR-D189; Toshiba Thailand Co., Ltd., Bangkok, Thailand) until further use in experimental procedures.

### 2.5. Lipid Peroxidation

Lipid peroxidation in semen samples was quantified using the thiobarbituric acid reactive substances assay, which measures the concentration of malondialdehyde (MDA), a key byproduct of membrane lipid peroxidation. MDA forms a chromogenic complex with thiobarbituric acid (TBA), resulting in an orange-pink color that is measurable via spectrophotometry [24].

Briefly, 250 µL of diluted semen (containing 250 × 10^6^ sperm/mL) was incubated with 0.25 mM ferrous sulfate and 0.25 mM ascorbic acid at 37 °C for 60 min. After incubation, 1 mL of 15% (*w*/*v*) trichloroacetic acid and 1 mL of 0.375% (*w*/*v*) TBA were added to the mixture. The samples were subsequently heated in a boiling water bath (100 °C) for 10 min. The reaction was terminated by rapid cooling in an ice water bath (4 °C), followed by centrifugation at 5000× *g* for 10 min at 4 °C. The absorbance of the supernatant was measured using a UV–visible spectrophotometer (Specord 250 Plus, Analytik Jena, Jena, Germany) at the appropriate wavelength (typically 532 nm). The concentration of MDA is expressed in micromolar units (µM/mL).

### 2.6. Experimental Design

#### 2.6.1. Experimental 1: Influence of Oxygen Exposure and Gentle Rotation on Rooster Sperm Quality During Chilled Storage at 5 °C for 24 h

This experiment was designed to evaluate the effects of oxygen exposure and sample rotation on the quality of rooster sperm during chilled storage. Two factors were tested: (1) oxygen exposure (aerobic vs. reduced-oxygen conditions), and (2) tube rotation (rotated vs. non-rotated). This resulted in four treatment groups: aerobic–rotated (A-R), aerobic–non-rotated (A-NR), reduced-O_2_–rotated (R-R), and reduced–O2–rotated (R-NR).

Oxygen exposure was manipulated by varying semen volume within 5 mL sterile microcentrifuge tubes (Eppendorf AG, Hamburg, Germany). A volume of 2.5 mL created a larger headspace, thereby representing aerobic conditions with increased oxygen availability, whereas a 5.0 mL volume filled the tube, minimizing headspace and simulating reduced oxygen conditions.

Following dilution, all samples were gradually cooled from 25 °C to 5 °C over a 60-min period and stored at 5 °C in a laboratory refrigerator. Samples assigned to the rotation group were gently inverted manually by tilting each tube three times at each 6 h interval throughout the 24 h storage period [8], whereas non-rotated samples remained stationary throughout the storage period.

Sperm quality parameters, including progressive motility, viability, MDA concentration, and semen pH, were assessed at three time points: immediately after cooling (0 h), and one hour after each rotation interval at 12 and 24 h of chilled storage. Each treatment combination was replicated five times. Based on sperm quality results, semen samples stored for 22 h under aerobic conditions, with and without rotation (A-R and A-NR), were selected for fertility testing, as these represented the optimal and control conditions within the factorial design. Both IGGKPh and 0.9% NaCl were included at this stage to compare an optimized extender with a commonly used field diluent. Semen samples stored under aerobic conditions (A-R and A-NR) for 22 h were selected for fertility testing using both extenders. Fertility testing was conducted in triplicate.

#### 2.6.2. Experimental 2; Effect of HEPES Buffer Supplementation on Rooster Sperm Quality During Simulated AI Handling

This experiment investigated the effects of HEPES buffer supplementation on the quality of rooster sperm during handling procedures that mimicked AI conditions. During AI, semen may be temporarily exposed to ambient temperature (~25 °C) or kept chilled at 5 °C, depending on field management practices. Two factors were tested: (1) HEPES buffer supplementation (present vs. absent), and (2) handling temperature during simulated AI (5 °C vs. 25 °C). This resulted in four treatment groups: no HEPES, handled at 5 °C (Control-5 °C), HEPES-supplemented extender, handled at 5 °C (HEPES-5 °C), no HEPES, handled at 25 °C (Control-25 °C), and HEPES-supplemented extender, handled at 25 °C (HEPES-25 °C).

All semen samples were previously stored under the optimal storage conditions identified in Experiment 1: an aerobic environment with gentle mechanical rotation. HEPES buffer was added to extenders at validated concentrations—15 mM for the 0.9% NaCl extender and 10 mM for the IGGKPh extender. These concentrations were selected based on titration experiments conducted prior to the main trial (see Supplementary Tables S1–S3), which demonstrated superior pH stability and preservation of sperm motility and viability at these levels. Control groups received the same extenders without HEPES.

After dilution, semen samples were handled differently based on extender type and experimental temperature. Samples diluted in IGGKPh were gradually cooled to 5 °C and stored under aerobic, rotated conditions for 24 h before being subjected to simulated AI handling. In contrast, semen diluted in 0.9% NaCl was not stored long-term; instead, it was immediately allocated to either chilled (5 °C) or ambient (25 °C) handling conditions for evaluation. Sperm quality was assessed at three time points: T0, immediately after 24 h of storage for IGGKPh or immediately after preparation for 0.9% NaCl; T30, after 30 min at the assigned handling temperature (5 °C or 25 °C); and T60, after 60 min of simulated AI handling. The evaluated parameters included progressive motility, viability, MDA concentration, and semen pH. Each treatment group was replicated five times. Fertility testing was performed using semen from all four treatment groups and replicated three times under standardized AI conditions.

### 2.7. Fertility Assessment

The fertility potential of rooster sperm was evaluated via AI. Inseminations were performed by depositing semen into the left side of the hens’ oviduct through the cloacal opening using a sterile tuberculin syringe. Each hen received 0.1 mL of semen containing approximately 150 × 10^6^ sperm per dose. Inseminations were conducted weekly between 3:00 p.m. and 5:00 p.m.

Eggs were collected daily, beginning on day 2 after the first insemination and continuing through day 8 following the final insemination. Collected eggs were stored in paper trays under controlled room temperature conditions (22–25 °C) and approximately 65–70% relative humidity for up to 7 days before incubation. Fertility was determined using candling on day 7 of incubation, and hatchability was assessed on day 21.

Fertility rates were calculated using the following formula: (total number of fertile eggs/total number of incubated eggs) × 100. The hatchability percentage was calculated using the following formula: (total number of chicks/total number of incubated eggs) × 100.

### 2.8. Statistical Analysis

To account for the fundamental compositional differences between the extenders, statistical analyses for NaCl and IGGKPh were conducted separately in both experiments. No cross-extender comparisons were performed.

In Experiment 1, a 2 × 2 factorial design (oxygen exposure × tube rotation) was employed within a completely randomized structure. Since semen quality was measured at three time points (0, 12, and 24 h), a two-way ANOVA was applied to assess time-dependent effects and interactions. The evaluated parameters included progressive motility (PMOT), viability, semen pH, and MDA concentration. Each treatment combination was replicated five times. For the fertility trial in Experiment 1, only semen stored under aerobic conditions (A-R and A-NR) with IGGKPh extender was tested. Fertility and hatchability data were analyzed using a generalized linear model (GLM) with a binomial distribution and a logit link function. The number of fertilized eggs per treatment was modeled as a proportion of the total eggs inseminated.

In Experiment 2, a split-plot design within a completely randomized framework was employed to investigate the effects of HEPES buffer supplementation (main plot factor: control vs. supplemented) and handling temperature (subplot factor: 5 °C vs. 25 °C). Each treatment group was assessed at three time points: T0 (immediately after storage or preparation), T30 (30 min), and T60 (60 min). These were treated as repeated measures in the model. Sperm quality parameters were analyzed using repeated-measures ANOVA, and each group was replicated five times. Fertility and hatchability data were analyzed using a GLM with binomial distribution as described for Experiment 1.

For both experiments, data normality (where applicable) was assessed using univariate analysis and the Shapiro–Wilk test. ANOVA procedures were conducted in SAS version 9.0 (PROC ANOVA or PROC MIXED), and GLM analysis was performed using PROC GENMOD (SAS Institute Inc., Cary, NC, USA). Where significant differences were detected, treatment means were compared using Tukey’s post hoc test. Statistical significance was set at *p* < 0.05.

## 3. Results

### 3.1. Experimental 1; Influence of Oxygen Exposure and Gentle Rotation on Rooster Sperm Quality During Chilled Storage at 5 °C for 24 h

**Figure 1:**
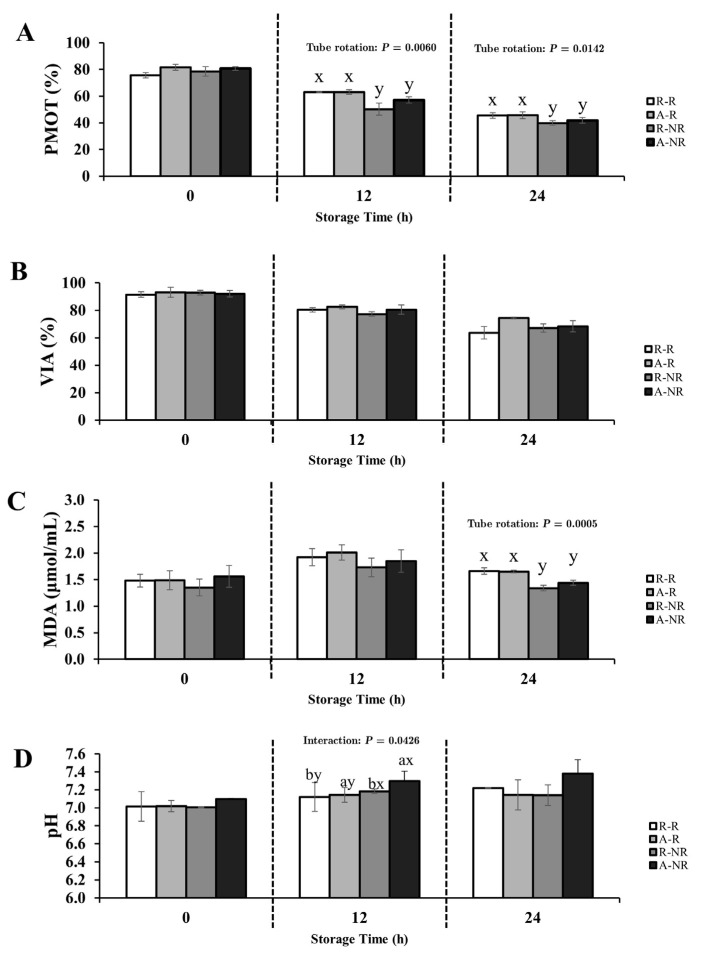
Effects of oxygen exposure (aerobic vs. reduced-O_2_) and tube rotation (rotated vs. non-rotated) on sperm quality parameters of Thai native rooster semen diluted with 0.9% NaCl extender during 24-h chilled storage at 5 °C. Panels show (**A**) progressive motility (PMOT), (**B**) viability (VIA), (**C**) malondialdehyde (MDA) concentration, and (**D**) semen pH. Treatment groups are defined as follows: R-R = reduced-O_2_–rotated; A-R = aerobic–rotated; R-NR = reduced-O_2_–non-rotated; A-NR = aerobic–non-rotated. Data are presented as mean ± SEM. *p*-values from two-way ANOVA (main effects and interaction) are annotated within each panel where statistically significant (*p* < 0.05). Different letters indicate significant pairwise differences due to (a, b) oxygen exposure and (x, y) tube rotation (*p* < 0.05).

**Figure 2:**
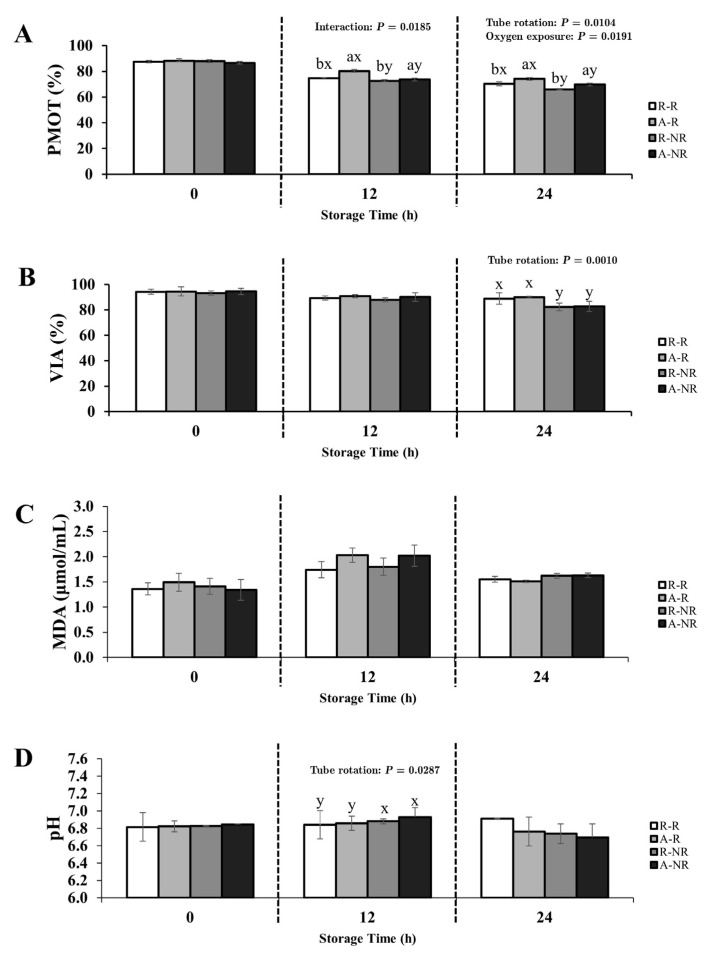
Effects of oxygen exposure and tube rotation on sperm quality parameters of Thai native rooster semen diluted with IGGKPh extender during 24-h chilled storage at 5 °C. Panels show (**A**) progressive motility (PMOT), (**B**) viability (VIA), (**C**) MDA concentration, and (**D**) semen pH. Treatment groups are defined as follows: R-R = reduced-O_2_–rotated; A-R = aerobic–rotated; R-NR = reduced-O_2_–non-rotated; A-NR = aerobic–non-rotated. Data are presented as mean ± SEM. *p*-values from two-way ANOVA (main effects and interaction) are annotated within each panel where statistically significant (*p* < 0.05). Different letters indicate significant pairwise differences due to (a, b) oxygen exposure and (x, y) tube rotation (*p* < 0.05).

The effects of oxygen exposure, gentle tube rotation, and their interaction on progressive motility (PMOT), viability (VIA), malondialdehyde (MDA) concentration, and pH were evaluated in Thai native rooster semen diluted with either 0.9% NaCl or IGGKPh extender. Full ANOVA results (main effects and interactions) are provided in Supplementary Table S4. Changes in sperm quality parameters over time are illustrated in Figure 1 and Figure 2, and statistically significant effects (*p* < 0.05) are annotated directly within the figure panels for improved clarity. Fertility outcomes are summarized in Table 1.

#### 3.1.1. Semen Diluted with 0.9% NaCl Extender

No significant interaction between oxygen exposure and tube rotation was observed for any parameter (*p* > 0.05). Oxygen exposure alone had no significant effect on PMOT, viability, MDA, or pH at any storage duration (*p* > 0.05). However, tube rotation had a significant main effect on motility and MDA. From 12 h onward, rotated samples showed significantly higher PMOT than non-rotated samples (*p* < 0.05), with improvement ranging from 6.01% to 12.67% (Figure 1A). This increase in motility was accompanied by a significant rise in MDA concentrations at 24 h in the rotated group (*p* < 0.05), with levels approximately 0.265 µmol/mL higher than in non-rotated samples (Figure 1C).

A significant interaction effect was observed for pH at 12 h (*p* < 0.05), with the A-NR group displaying higher pH values (7.30; Figure 1D).

No significant effects were observed on sperm viability at any time point (*p* > 0.05; Figure 1B).

#### 3.1.2. Semen Diluted with IGGKPh Extender

Oxygen exposure and tube rotation significantly affected PMOT beginning at 12 h of storage (*p* < 0.05). The A-R group maintained the highest motility values throughout the storage period, reaching 80.18% at 12 h and 74.23% at 24 h (Figure 2A).

Tube rotation also had a significant effect on viability at 24 h (*p* < 0.05), with rotated samples averaging 89.48% compared with 82.53% in non-rotated samples (Figure 2B). No significant interaction between oxygen exposure and rotation was detected for MDA concentration (*p* > 0.05; Figure 2C).

At 12 h, pH values were significantly influenced by rotation (*p* < 0.05), with non-rotated samples showing slightly higher pH (6.91) than rotated samples (6.85; Figure 2D).

#### 3.1.3. Fertility Outcomes

For the fertility test, semen stored under aerobic conditions for 22 h, with and without rotation, was used for insemination. Due to the 0% fertility of samples diluted in 0.9% NaCl, this group was excluded from statistical comparison. This is likely to reflect the extender’s insufficient buffering and energy support, as further discussed in Section 4.1 (Extender Comparison: 0.9% NaCl vs. IGGKPh). Therefore, only IGGKPh-diluted groups were included in the fertility analysis.

Fertility rates differed significantly between treatment groups (*p* < 0.05). The A-R group exhibited significantly higher fertility (91.77 ± 3.06%) compared with A-NR (75.32 ± 6.70%; *p* < 0.05). Hatchability did not differ significantly between groups (*p* > 0.05).

### 3.2. Experimental 2; Effect of HEPES Buffer Supplementation on Rooster Sperm Quality During Simulated AI Handling

**Figure 3:**
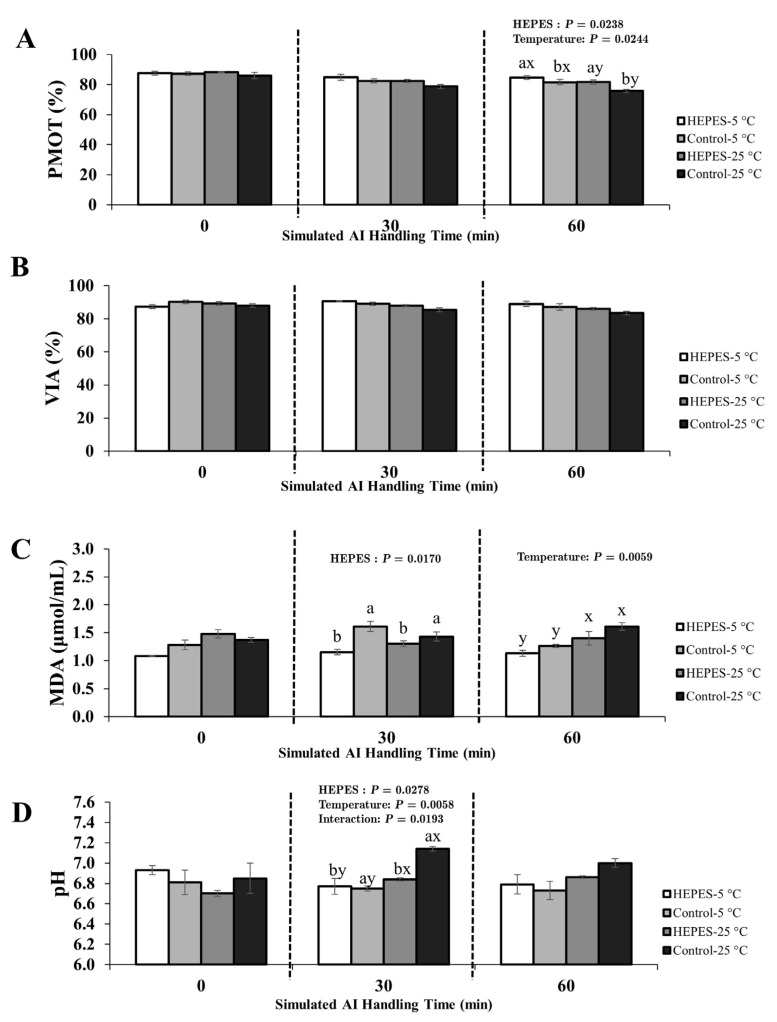
Effects of HEPES buffer supplementation and handling temperature on sperm quality parameters of Thai native rooster semen diluted with 0.9% NaCl extender during simulated artificial insemination (AI) handling at 0, 30, and 60 min after dilution. Panels show (**A**) progressive motility (PMOT), (**B**) viability (VIA), (**C**) malondialdehyde (MDA) concentration, and (**D**) pH. Treatment groups are defined as follows: HEPES-5 °C = HEPES-supplemented, handled at 5 °C; Control-5 °C = no HEPES, handled at 5 °C; HEPES-25 °C = HEPES-supplemented, handled at 25 °C; Control-25 °C = no HEPES, handled at 25 °C. Values represent mean ± SEM. *p*-values from two-way ANOVA (main effects and interaction) are annotated within each panel where statistically significant (*p* < 0.05). Different letters indicate significant pairwise differences due to (a, b) HEPES supplementation and (x, y) handling temperature (*p* < 0.05).

**Figure 4:**
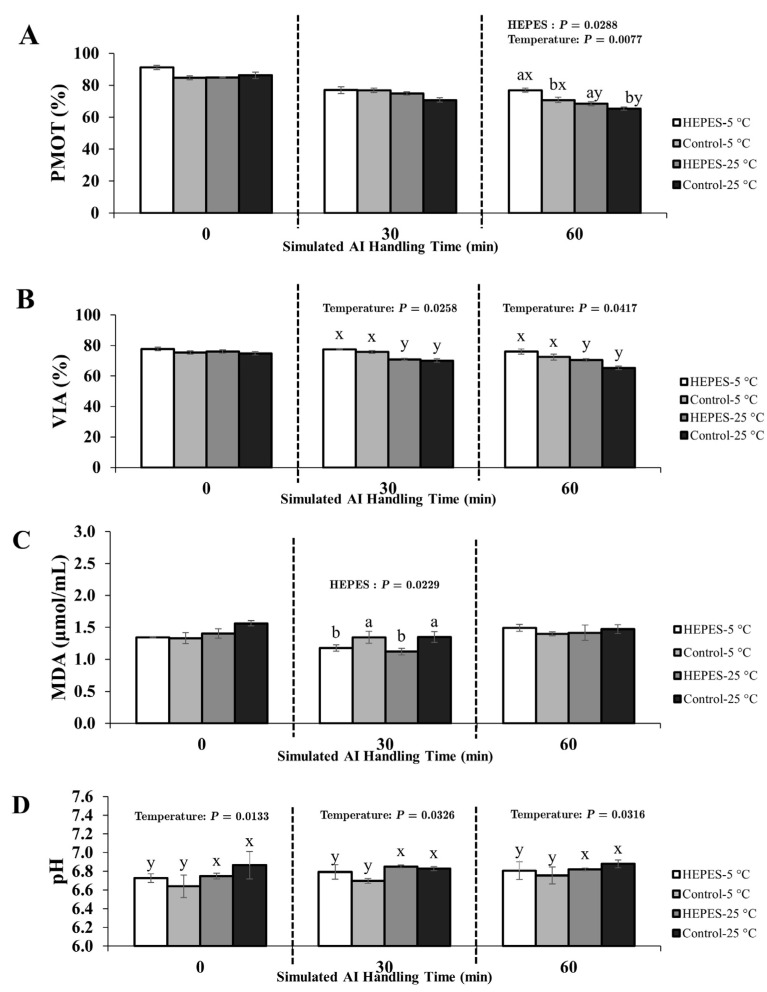
Effects of HEPES buffer supplementation and handling temperature on sperm quality parameters of Thai native rooster semen diluted with IGGKPh extender during simulated artificial insemination (AI) handling at 0, 30, and 60 min after 22 h of chilled storage. Panels show (**A**) progressive motility (PMOT), (**B**) viability (VIA), (**C**) malondialdehyde (MDA) concentration, and (**D**) pH. Treatment groups are defined as follows: HEPES-5 °C = HEPES-supplemented, handled at 5 °C; Control-5 °C = no HEPES, handled at 5 °C; HEPES-25 °C = HEPES-supplemented, handled at 25 °C; Control-25 °C = no HEPES, handled at 25 °C. Values represent mean ± SEM. *p*-values from two-way ANOVA (main effects and interaction) are annotated within each panel where statistically significant (*p* < 0.05). Different letters indicate significant pairwise differences due to (a, b) HEPES supplementation and (x, y) handling temperature (*p* < 0.05).

The effects of HEPES supplementation, handling temperature, and their interaction on PMOT, viability, MDA concentration, and pH were evaluated at three post-storage time points using Thai native rooster semen diluted with either 0.9% NaCl or IGGKPh extender. Full ANOVA results (main effects and interactions) are provided in Supplementary Table S5. Mean values for each treatment are presented in Figure 3 and Figure 4, and statistically significant effects (*p* < 0.05) are annotated directly within the figure panels for improved clarity. Fertility outcomes are shown in Table 2.

#### 3.2.1. Semen Diluted with 0.9% NaCl Extender

At 0 min (T0), no significant main or interaction effects were observed for any sperm quality parameters (*p* > 0.05).

At 30 min (T30), HEPES supplementation had a significant effect on MDA concentration (*p* < 0.05), with higher levels in the control group (1.61 µmol/mL) compared with the HEPES group (1.15 µmol/mL; Figure 3C). A significant HEPES × temperature interaction was observed for pH (*p* < 0.05), with the highest value recorded in the control-25 °C group (7.14; Figure 3D). No significant differences were found for PMOT or viability at this time point (*p* > 0.05).

At 60 min (T60), both HEPES and temperature significantly affected PMOT (*p* < 0.05). HEPES-supplemented samples showed higher PMOT (84.72%) than controls (81.61%), and samples held at 5 °C exhibited higher motility (83.17%) than those at 25 °C (78.69%; Figure 3A). MDA levels remained higher in samples exposed to 25 °C, and no significant differences in viability were observed (*p* > 0.05; Figure 3B).

#### 3.2.2. Semen Diluted with IGGKPh Extender

At T0, no significant effects of HEPES supplementation or the HEPES × temperature interaction were observed for any parameter (*p* > 0.05). However, temperature had a significant effect on pH (*p* < 0.05), with higher values in samples stored at 25 °C (Figure 4D).

At T30, HEPES supplementation significantly reduced MDA concentration (1.14 µmol/mL vs. 1.34 µmol/mL in controls; *p* < 0.05; Figure 4C). Temperature had a significant effect on viability (*p* < 0.05), with higher viability in the 5 °C group (76.64%) compared with 25 °C (70.46%; Figure 4B). pH also varied significantly with temperature (*p* < 0.05), showing a gradual increase at 25 °C. No interaction effects were observed (*p* > 0.05).

At T60, both HEPES supplementation and temperature had significant effects on PMOT (*p* < 0.05). The HEPES-5 °C group exhibited the highest motility (76.88%). The HEPES-25 °C (68.50%) and Control-5 °C (70.75%) groups did not differ significantly (*p* > 0.05). Viability remained higher in the 5 °C group (74.25%) compared with 25 °C (65.41%; *p* < 0.05). No significant differences were detected in MDA concentration or pH at this time point.

#### 3.2.3. Fertility Outcomes

Table 2 summarizes the effects of HEPES supplementation and handling temperature on fertility and hatchability in Thai native rooster semen extended with 0.9% NaCl or IGGKPh.

For the NaCl extender, fertility rates differed significantly among treatment groups (*p* < 0.05). The HEPES-5 °C group yielded the highest fertility (82.80 ± 6.48%), followed by HEPES-25 °C (79.50 ± 3.83%). Both groups showed significantly higher fertility than the control group at 25 °C (57.09 ± 4.45%). No significant differences in hatchability were observed (*p* > 0.05).

For the IGGKPh extender, fertility rates also differed significantly (*p* < 0.05). The HEPES-5 °C group showed the highest fertility (79.59 ± 0.41%), significantly higher than the other treatments. The Control-5 °C group (69.62 ± 0.38%), HEPES-25 °C (64.92 ± 1.76%), and control-25 °C (66.36 ± 1.65%) did not differ significantly from each other (*p* > 0.05). Hatchability was not significantly affected by treatment with either extender (*p* > 0.05).

## 4. Discussion

This study evaluated a range of physical and biochemical strategies aimed at enhancing the short-term preservation and fertilizing capacity of rooster semen under chilled storage and artificial insemination handling conditions. By systemically examining the effects of extender composition, oxygen exposure, tube rotation, and HEPES buffer supplementation, the findings provide novel insights into how storage environment and extender formulation influence sperm viability, motility, oxidative stability, and, ultimately, fertility outcomes. The following discussion considers each of these factors in turn and explores their physiological implications, practical applications, and potential for further optimization.

### 4.1. Extender Comparison: 0.9% NaCl vs. IGGKPh

Although direct statistical comparisons were not made between the two extenders, the present findings clearly demonstrate their differing capacities to preserve rooster sperm quality during chilled storage. Semen diluted with 0.9% NaCl exhibited a sharp decline in progressive motility and viability after 12 h, highlighting its limitation for prolonged storage. This decline is likely to result from the absence of key metabolic substrates such as glucose and inositol, which are essential for sustaining sperm energy metabolism and motility [25]. While tube rotation modestly improved PMOT by promoting fluid homogeneity, it did not sustain sperm function beyond 24 h.

MDA levels in NaCl samples remained low, indicating that oxidative stress was not the primary driver of sperm deterioration. Rather, the loss of motility and viability appears related to energy depletion due to the extender’s minimal composition. Fertility outcomes support this conclusion; semen stored in NaCl for 22–24 h resulted in 0% fertilization. These findings align with previous reports that saline-based extenders lack essential components for preserving fertilizing capacity during extended storage [26,27]. While NaCl remains widely used in low-resource settings, the present data indicate it is unsuitable for storage beyond 12 h when fertility preservation is a goal.

In contrast, IGGKPh maintained higher sperm quality throughout 24 h of chilled storage. PMOT remained above 70% and viability close to or above 90%, suggesting effective preservation. The inclusion of glucose and inositol in IGGKPh likely supports mitochondrial ATP production, contributing to sustained motility and membrane integrity [28,29]. However, a moderate increase in MDA was noted in the rotated, aerobic IGGKPh group at 24 h, indicating some lipid peroxidation, potentially due to elevated metabolic activity during storage. This finding aligns with prior evidence suggesting that avian sperm are particularly susceptible to oxidative stress due to their high content of polyunsaturated fatty acids [30,31]. While IGGKPh is effective for short-term storage, it may benefit from antioxidant supplementation to limit ROS-related damage during prolonged storage periods.

### 4.2. The Influence of Oxygen Exposure on Rooster Sperm Preservation

Oxygen exposure is often considered detrimental to sperm preservation due to its role in promoting ROS formation, which can impair membrane integrity and sperm function [13,32,33]. However, the present study found that aerobic storage improved sperm quality in IGGKPh-diluted samples, indicating a potentially species-specific metabolic adaptation in avian sperm.

Unlike mammalian sperm, which rely mainly on glycolysis for ATP production [34,35], avian sperm utilize both aerobic and anaerobic metabolic pathways [36]. The enhanced motility and viability observed under aerobic conditions, particularly with IGGKPh, suggest that rooster sperm can effectively harness oxidative phosphorylation when provided with energy substrates, such as glucose and inositol, both of which are present in IGGKPh.

By contrast, semen stored in 0.9% NaCl, which lacks such substrates, did not benefit from oxygen exposure. This finding reinforces the importance of extender composition in determining whether oxygen exposure acts as a metabolic enhancer or a source of oxidative damage.

Although oxygen supports ATP generation, it also increases the potential for oxidative stress. This was reflected in higher MDA concentrations at 24 h in the aerobic-rotated IGGKPh group. Nonetheless, motility and viability remained high, indicating that during short-term storage, the benefits of enhanced metabolism can outweigh the damage caused by ROS. These findings suggest that oxygen exposure may be beneficial when combined with nutrient-rich extenders, but it may have adverse effects in metabolically inert media or under prolonged storage conditions.

### 4.3. The Impact of Tube Rotation on Sperm Quality

Tube rotation was assessed for its role in minimizing sedimentation and promoting homogeneity during semen storage. Results showed that rotation significantly improved progressive motility, particularly in IGGKPh-diluted samples at 12 and 24 h under aerobic conditions. A smaller but significant improvement in viability was also observed in IGGKPh at 24 h. These findings suggest that gentle rotation enhances sperm quality, especially when used with metabolically supportive extenders.

Three mechanisms may explain the observed benefits of rotation during semen storage. First, rotation maintains uniform sperm suspension and extender distribution, ensuring consistent exposure to nutrients and pH buffers [37]. Second, rotation may facilitate gas exchange within the storage tube. In glucose-based extenders like IGGKPh, this can improve oxygen availability for mitochondrial respiration and promote CO_2_ dissipation, thereby stabilizing pH and minimizing acid-base fluctuations [38]. Third, rotation may help disperse ROS more evenly, reducing localized oxidative hotspots and limiting damage to sperm subpopulations [17].

Despite these advantages, rotation also increased MDA concentrations in NaCl-diluted samples at 24 h, indicating elevated oxidative stress. Although this did not improve viability, motility still benefited, suggesting some short-term advantage even in nutrient-poor media. In IGGKPh, the combination of oxygen exposure and rotation appeared to stimulate metabolic activity, which may have increased oxidative processes, as reflected by higher MDA levels by 24 h.

These results highlight a trade-off; while rotation supports motility and viability, it may also elevate oxidative stress, particularly under aerobic conditions. This aligns with prior studies showing that increased mitochondrial activity in avian sperm can surpass intrinsic antioxidant defenses, leading to lipid peroxidation [39,40]. Thus, rotation should ideally be paired with antioxidant supplementation or oxygen regulation during extended storage to balance metabolic enhancement with oxidative protection.

### 4.4. The Role of HEPES Buffer Supplementation in Enhancing Sperm Preservation During AI Handling

In poultry AI, chilled semen storage at 5 °C is commonly used to suppress metabolic activity and limit oxidative stress. However, during field procedures, semen often undergoes brief warming to ambient temperature (~25 °C), which may impair sperm function if not properly buffered.

Rooster sperm are known to survive short exposure to room temperature, typically for 1–2 h, but prolonged exposure or higher temperatures (≥40 °C) compromise motility and mitochondrial function [41]. Previous studies also show increased oxygen consumption at moderate temperatures, with sharp declines in oxidative capacity beyond 41 °C [42]. These findings highlight the need for extenders that provide thermal and oxidative stability during AI handling.

In this study, HEPES supplementation significantly improved PMOT at certain time points (notably at 60 min) and reduced MDA levels during simulated AI handling at both 5 °C and 25 °C. These protective effects were consistent across both NaCl and IGGKPh extenders, demonstrating their broad compatibility.

HEPES is a zwitterionic buffering agent with a stable pKa of 7.5 and effective pH regulation across 5–55 °C [43]. Treated groups exhibited more stable pH levels and higher viability, particularly at 25 °C. In contrast, NaCl samples without HEPES exhibited greater pH elevation at ambient temperature (up to 7.14), likely due to ammonia accumulation or calcium efflux, processes that disrupt membrane potential and sperm function [44,45].

Fertility outcomes were also improved in HEPES-supplemented groups, reinforcing its functional benefit during short-term storage and handling. While hatchability remained unaffected, the increase in fertility suggests that HEPES may enhance the pre-insemination quality of sperm, especially in field conditions where temperature fluctuations are difficult to control. Similar protective effects have been reported in mammalian sperm under thermal and oxidative stress [21,22].

Rooster sperm function is optimized at pH 6.8–7.1 [46]. While this range was not strictly maintained in all treatments, HEPES helped keep values closer to this optimal range under both chilled and ambient handling, potentially reducing oxidative damage, as supported by lower MDA levels in some conditions.

In summary, HEPES buffer can enhance sperm quality during AI handling by stabilizing pH, mitigating oxidative stress, and maintaining motility under thermal variation. Its use is especially beneficial in field conditions where temperature control is limited, and it may represent a practical improvement to existing semen extender protocols.

## 5. Conclusions

This study systematically evaluated physical and biochemical strategies, including oxygen exposure, tube rotation, and HEPES buffering, to improve rooster semen preservation during chilled storage and AI handling. The results emphasize the need to align extender composition, environmental factors, and buffering capacity with the metabolic characteristics of avian sperm.

Oxygen exposure enhanced sperm quality in roosters, particularly when combined with nutrient-rich extenders like IGGKPh. While tube rotation improved sample homogeneity and performance, increased oxidative stress was observed under aerobic, rotated conditions at 24 h.

HEPES supplementation helped to stabilize pH in several conditions and reduced oxidative damage during post-storage handling. These effects were observed in both NaCl and IGGKPh extenders, though the magnitude varied depending on temperature and handling time. NaCl-based extenders, although widely used in field settings due to accessibility, were suitable only for short-term use and benefited substantially from HEPES inclusion.

Overall, IGGKPh provided the most consistent preservation for 24 h chilled storage, especially under aerobic and rotated conditions, making it a suitable candidate for centralized AI programs in poultry. These findings support practical improvements in semen preservation that are adaptable to both laboratory protocols and on-farm conditions.

Future research should explore integrating these physical and biochemical strategies with updated extender technologies such as nanoemulsions, colloidal carriers, and gel-based systems to further enhance sperm viability and fertility outcomes under field conditions.

## Figures and Tables

**Table 1:** Fertility outcomes of Thai native rooster semen stored under aerobic conditions with or without tube rotation using the 0.9% NaCl and IGGKPh extenders.

Parameter	Extender	Aerobic-Rotated	Aerobic-Non-Rotated	*p*-Value
No. of eggs		448	403	
Fertility (%)	0.9%NaCl	N/A	N/A	-
	IGGKPh	91.77 ± 3.06 ᵃ	75.32 ± 6.70 ᵇ	0.021
Hatchability (%)	0.9%NaCl	N/A	N/A	-
	IGGKPh	91.58 ± 3.28	91.58 ± 4.06	0.489

Abbreviation: Data for the 0.9% NaCl group were excluded due to 0% fertility and hatchability across all replicates. N/A = not analyzed due to 0% fertility. Values represent mean ± standard error of the mean (SEM). Different superscript letters within rows (^a, b^) indicate significant differences between treatments (*p* < 0.05).

**Table 2:** Effects of HEPES buffer supplementation and handling temperature on fertility and hatchability of Thai native rooster semen extended with 0.9% NaCl or IGGKPh.

Treatment	No. of Eggs	Parameter			
		0.9% NaCl		IGGKPh	
		Fertility (%)	Hatchability (%)	Fertility (%)	Hatchability (%)
Control-5 °C	358	69.85 ± 2.59 ^ab^	74.58 ± 4.58	69.62 ± 0.38 ^b^	83.86 ± 3.86
HEPES-5 °C	400	82.80 ± 6.48 ^a^	78.75 ± 1.25	79.59 ± 0.41 ^a^	93.86 ± 6.14
Control-25 °C	442	57.09 ± 4.45 ^b^	75.66 ± 1.09	66.36 ± 1.65 ^b^	90.02 ± 2.30
HEPES-25 °C	437	79.50 ± 3.83 ^a^	73.63 ± 3.04	64.92 ± 1.76 ^b^	83.15 ± 4.58
*p*-Value		0.0033	0.621	0.0037	0.3813

Abbreviation: Semen samples were stored for 22 h at 5 °C and subsequently incubated for 60 min at either 5 °C or 25 °C to simulate artificial insemination (AI) handling. Four treatment groups were compared: Control (no HEPES supplementation) and HEPES-supplemented, each incubated at 5 °C (chilled) or 25 °C (ambient). Data are presented separately for semen extended in 0.9% NaCl and IGGKPh. Values are mean ± standard error of the mean (SEM). Different superscript letters (^a, b^) within the same column indicate significant differences (*p* < 0.05).

## Data Availability

Data are available upon request to the corresponding author.

## Supplementary Materials

The following supporting information can be downloaded at: https://www.mdpi.com/article/10.3390/ani15162391/s1, Table S1: Effect of HEPES concentration on pH stability in NaCl and IGGKPh extenders during incubation at 5 °C and 25 °C. Table S2: Effect of HEPES supplementation on rooster sperm motility and viability in NaCl extender during chilled storage at 5 °C. Table S3: Effect of HEPES supplementation on rooster sperm motility and viability in IGGKPh extender during chilled storage at 5 °C. Table S4: Two-way ANOVA results for Experiment 1 (oxygen × rotation). Table S5: Two-way ANOVA results for Experiment 2 (HEPES × temperature).
