# Supplementary material for: Optimizing Rooster Semen Preservation: Effect of Oxygen Exposure, Sample Rotation, and HEPES Buffer Supplementation
Source: Animals (Basel). 2025 Aug 14;15(16):2391. doi: 10.3390/ani15162391 (PMC12383207; doi:10.3390/ani15162391)
Supplement: Supplementary file 1 [file animals-15-02391-s001.zip › animals-3775284-supplementary.pdf]

Supplementary Table S1. Effect of HEPES Concentration on pH Stability in NaCl and IGGKPh Extenders during Incubation at 5 °C and 25 °C

**Extender**	**HEPES (mM)**	**0 min**		**30 min**		**60 min**	
		**5 °C**	**25 °C**	**5 °C**	**25 °C**	**5 °C**	**25 °C**
**0.9% NaCl**	5	6.55 ± 0.005	6.53 ± 0.005	6.57 ± 0.008	6.56 ± 0.018	6.59 ± 0.008	6.54 ± 0.012
	10	6.60 ± 0.011	6.60 ± 0.005	6.61 ± 0.008	6.62 ± 0.008	6.63 ± 0.006	6.61 ± 0.012
	15	6.78 ± 0.005	6.76 ± 0.008	6.77 ± 0.005	6.76 ± 0.005	6.78 ± 0.005	6.76 ± 0.005
	20	6.75 ± 0.005	6.71 ± 0.008	6.71 ± 0.005	6.72 ± 0.003	6.77 ± 0.058	6.72 ± 0.014
**IGGKPh**	5	7.00 ± 0.005	6.98 ± 0.003	6.98 ± 0.005	6.99 ± 0.005	7.00 ± 0.006	6.99 ± 0.005
	10	6.99 ± 0.005	6.97 ± 0.008	6.98 ± 0.003	6.98 ± 0.003	6.99 ± 0.008	6.74 ± 0.225
	15	6.99 ± 0.003	6.97 ± 0.005	7.00 ± 0.005	6.99 ± 0.003	6.99 ± 0.003	6.99 ± 0.008
	20	6.99 ± 0.003	6.98 ± 0.003	6.67 ± 0.328	6.97 ± 0.003	7.00 ± 0.010	6.99 ± 0.003

Mean ± SEM of pH values measured at 0, 30, and 60 min during incubation at 5 °C and 25 °C. HEPES-supplemented NaCl and IGGKPh were evaluated to determine the optimal buffering capacity suitable for chilled semen handling.

Supplementary Table S2. Effect of HEPES Supplementation on Rooster Sperm Motility and Viability in 0.9% NaCl Extender at 5 °C

Storage Time (h)	Parameter	Control	5 mM	10 mM	15 mM	20 mM	SEM
**0**	PMOT (%)	79.94	79.25	81.31	84.82	80.91	1.96
	VIA (%)	89.76	88.74	93.35	94.06	89.80	1.20
**12**	PMOT (%)	54.09	63.49	61.84	74.80	69.98	4.04
	VIA (%)	75.37	73.58	76.43	79.27	70.01	1.04
**24**	PMOT (%)	51.47	44.31	42.41	46.11	50.52	3.85
	VIA (%)	63.97	61.90	62.07	64.09	62.61	0.33

Mean (%) of progressive motility (PMOT) and viability (VIA) of rooster sperm stored in 0.9% NaCl extender supplemented with different concentrations of HEPES, measured at 0, 12, and 24 h. SEM: Standard error of the mean.

Supplementary Table S3. Effect of HEPES Supplementation on Rooster Sperm Motility and Viability in IGGKPh Extender at 5 °C

Storage Time (h)	Parameter	Control	5 mM	10 mM	15 mM	20 mM	SEM
**0**	PMOT (%)	79.95	74.22	80.80	79.81	73.55	5.75
	VIA (%)	94.15	93.56	95.58	92.66	88.86	0.39
**12**	PMOT (%)	75.17	75.38	79.47	75.70	73.85	3.13
	VIA (%)	85.85	84.34	89.32	81.20	79.95	1.71
**24**	PMOT (%)	68.87	70.33	75.01	72.66	68.29	3.59
	VIA (%)	77.78	77.98	82.28	78.34	75.72	1.02

Mean (%) of progressive motility (PMOT) and viability (VIA) of rooster sperm stored in IGGKPh extender supplemented with different concentrations of HEPES, measured at 0, 12, and 24 h. SEM: Standard error of the mean.

Supplementary Table S4. Summary of analysis of variance (ANOVA) for the effects of oxygen exposure, tube rotation, and their interaction on sperm quality parameters of Thai native rooster semen stored with either 0.9% NaCl or IGGKPh extender at 5°C.

Extender	Time	Effect	PMOT	VIA	MDA	pH
0.9% NaCl	0 h	Oxygen exposure	NS	NS	NS	NS
		Tube rotation	NS	NS	NS	NS
		Oxygen × Rotation	NS	NS	NS	NS
	12 h	Oxygen exposure	NS	NS	NS	NS
		Tube rotation	**	NS	NS	NS
		Oxygen × Rotation	NS	NS	NS	*
	24 h	Oxygen exposure	NS	NS	NS	NS
		Tube rotation	*	NS	**	NS
		Oxygen × Rotation	NS	NS	NS	NS
IGGKPh	0 h	Oxygen exposure	NS	NS	NS	NS
		Tube rotation	NS	NS	NS	NS
		Oxygen × Rotation	NS	NS	NS	NS
	12 h	Oxygen exposure	**	NS	NS	NS
		Tube rotation	**	NS	NS	*
		Oxygen × Rotation	*	NS	NS	NS
	24 h	Oxygen exposure	*	NS	NS	NS
		Tube rotation	*	**	NS	NS
		Oxygen × Rotation	NS	NS	NS	NS

Abbreviation: PMOT = progressive motility; VIA = viability; MDA = malondialdehyde; NS = not significant ($P > 0.05$); * = significant ($P < 0.05$); ** = highly significant ($P < 0.01$). Factor definitions: "Oxygen exposure" refers to aerobic vs. reduced-oxygen conditions; "Tube rotation" refers to rotated vs. non-rotated treatments.

Supplementary Table S5. Analysis of variance (ANOVA) results for the effects of HEPES buffer supplementation, handling temperature, and handling time on sperm quality parameters of Thai native rooster semen diluted with 0.9% NaCl or IGGKPh extender.

Extender	Time	Effect	PMOT	VIA	MDA	pH
0.9% NaCl	0 min	HEPES supplementation	NS	NS	NS	NS
		Temperature	NS	NS	NS	NS
		HEPES × Temperature	NS	NS	NS	NS
	30 min	HEPES supplementation	NS	NS	*	*
		Temperature	NS	NS	NS	**
		HEPES × Temperature	NS	NS	NS	*
	60 min	HEPES supplementation	*	NS	NS	NS
		Temperature	*	NS	**	NS
		HEPES × Temperature	NS	NS	NS	NS
IGGKPh	0 min	HEPES supplementation	NS	NS	NS	NS
		Temperature	NS	NS	NS	*
		HEPES × Temperature	NS	NS	NS	NS
	30 min	HEPES supplementation	NS	NS	*	NS
		Temperature	NS	*	NS	*
		HEPES × Temperature	NS	NS	NS	NS
	60 min	HEPES supplementation	*	NS	NS	NS
		Temperature	**	*	NS	*
		HEPES × Temperature	NS	NS	NS	NS

Abbreviation: Handling time refers to simulated artificial insemination (AI) at 0, 30, and 60 min after 22 h of chilled storage. ANOVA was performed separately for each extender type (0.9% NaCl and IGGKPh). HEPES supplementation: comparison between semen with vs. without HEPES buffer. Temperature: comparison between samples maintained at 5 °C or 25 °C during simulated AI.
